# The Brooklyn Dental Infirmary

**Published:** 1870-05

**Authors:** Geo. A. Mills


### THE BROOKLYN DENTAL INFIRMARY.

BY GEO. A. MILLS.

This institution had its origin in the Brooklyn Dental Society, and we feel (with a little pride) that it is second to no other local Dental society. It has between 40 and 50 members—good working men. All our meetings are especially characterized by harmony. We have no time for personal jealousies. We have been coming up since the day of our organization as a society. Which will be three years next October. Our meetings are held once in two weeks, itinerating from office to office. We average about thirty at each meeting. We are all glad to see each other, and are quite loth to part at the appointed hour for adjournment, which is ten o'clock, P. M. At nearly all our meetings we retire from labor to refreshments. Although we provide but little in variety; the avidity with which it is cared for shows a just appreciation. And allow me to say, if this comes to the notice of any Dental society that is fainting by the way, adopt our method. Have something to work for; and after you have worked hard from 8 till 10 P. M., take something, if no more than a ham sandwich. Try it.

Very soon after the organization of our society, a movement was made to establish a Dental Infirmary. A commencement was soon made, or we might say, the society entered upon the project, and made a beginning at the rooms of the Homœopathy Dispensary in Brooklyn. And through the kindness of the officers of that institution, we tenderly took care of the embryo under their roof. When the time had come for its deliverance, we sought a home for the baby. Some delay was made, but soon fears began to arise that we would lose it through neglect. But soon the travaling come, with full pains, and a delivery was made to the society, by a unanimous vote. A house was secured, by the generous contributions of some of the members, and at noon, January 10th, 1870, the child was christened, in the presence of gathered members of the society and of the Brooklyn press. And with many joint wishes and congratulatory speeches it was named the Brooklyn Dental Infirmary.

Since that day all goes well, and a growing interest is being manifested, not only by the society, but the public. This institution is managed by a clinical committee to be appointed by the Society each year. From January to May the expenses were borne by the society in part. To warrent the society in renewing the lease for a year, efforts were made by members to secure funds from the citizens, and being quite successful the rooms have been leased for one year from the first of May, 1870.

We invite all Dentists especially to visit us, and we assure you a cordial welcome. Special clinics have now been instituted on the first Wednesday of each month, from 2 till 4 P. M.

All practical Dentists will at once see the benefits accruing from this associated effort, both in the daily operations at the infirmary (which are clinics) and these special clinics.

This somewhat long and detailed account I have been moved to give, hoping that it might not only encourage but stimulate increased energy in our profession.

We have first been greatly encouraged in a pecuniary point by an appropriation from the State of $1,500 00, which will do much to improve the internal condition of our infirmary.

